# Workshop Highlights

**DOI:** 10.6028/jres.101.041

**Published:** 1996

**Authors:** Vicky Lynn Karen, Alan Mighell

The ongoing computer revolution is causing changes in all crystallographic data activities and is blurring distinctions between what have traditionally been independent efforts. As computer technology removes barriers in data storage, retrieval, and networking, unprecedented opportunities arise to achieve the dream of all crystallographers: easy access to *all* crystallographic data. It was timely, therefore, to speculate on how the future would evolve and how to direct activities towards this dream. During formal and informal discussions, many tough and provocative questions were asked that focused on the relevant issues including the quality of data, search software, political and economic concerns, and cooperation among users and producers.

One topic stressed was the importance of quality throughout all stages of the data flow including collection, publication, and incorporation into a database. Who would be the guardian of quality in the various stages of the data flow? With respect to quality control across different journals, one attendee stated, “We might inquire whether other journals would be willing (or could be persuaded) to impose quality controls similar to the way in which Acta Crystallographica checks structural data in papers submitted to its journal.” Another, more complex issue, centers around the incorporation of unpublished data directly into a database without compromising quality.

A second major focus was software tools. Many users emphasized that use and acceptance of crystallographic databases by the general scientific community is directly related to the availability and quality of the search tools. The discussion identified two areas of great need: searching across different crystallographic databases and the development of new software tools. One attendee asked, “Is it possible to devise software that will make searches possible across more than one database? For example, a study of hydrogen bonding requires a search of both inorganic and organic databases.” Another enquired about “the feasibility of a common query program (or language) for structural databases.” With regard to software tools, many attendees repeated the need for new scientific algorithms to seek and recognize structural patterns, motifs, structure types, etc.

A third theme was related to access and availability of all the information. Among the questions raised were: how data will be made available to the user community, and “Who will be the power brokers in the future technologies? Will it be the companies that have privatized all existing physical data and control access to it? Should not all of the crystallographic data be freely obtained by the user community, much the way we use libraries?” Further discussion recognized the economic value of structural data and the costs of formal data activities.

Issues such as these demonstrate the need among the representatives of the various data activities for future meetings to foster a continuing dialog. In addition, many attendees emphasized the need for much greater cooperation among the data centers, among the journals and the data centers, and among users and the data centers.

The awareness of these problems represents a major step toward finding solutions in this period of rapid technological change. We look forward to a future with continued and enhanced cooperation in the crystallographic community.

